# Infantile Vomiting Is Not Always What You Think: The Importance of a Broad Differential Diagnosis

**DOI:** 10.7759/cureus.104637

**Published:** 2026-03-03

**Authors:** Karlee B Schultz-Davidson, Lydia Hanson, Lucas McKnight, Pablo J Sanchez

**Affiliations:** 1 Internal Medicine-Pediatrics, The Ohio State University College of Medicine, Columbus, USA; 2 Internal Medicine-Pediatrics, The Ohio State University Wexner Medical Center and Nationwide Children's Hospital, Columbus, USA; 3 Internal Medicine, The Ohio State University Wexner Medical Center and Nationwide Children's Hospital, Columbus, USA; 4 Infectious Disease, Nationwide Children’s Hospital, Columbus, USA

**Keywords:** bilious vomiting, combination therapy of vancomycin and daptomycin, hepatic abscess, infant, mrsa bacteremia

## Abstract

Characterized by its green or yellowish color, bilious vomiting in an infant is often an indicator of an anatomic abnormality such as intestinal malrotation with volvulus, duodenal atresia/stenosis, or obstruction. Some infectious causes include acute gastroenteritis, norovirus, parasitic infections, and necrotizing enterocolitis (NEC). Here, we present an unusual case of an infant with bilious vomiting due to methicillin-resistant *Staphylococcus aureus* (MRSA) bacteremia with hepatic abscess. While rare, this case highlights the importance of adding this diagnosis to the differential as MRSA continues to be problematic in both the community and hospital.

## Introduction

While both a commensal bacterium and a human pathogen, *Staphylococcus aureus* is a leading cause of both community and hospital-acquired infections [1]. The most common type of *S. aureus* infection involves the skin and subcutaneous tissues; invasive infections such as bacteremia are a serious threat [1]. In fact, *S. aureus* remains the most common cause of invasive bacterial infections in children [2]. With the rise of methicillin-resistant *S. aureus* (MRSA), there is a level of complexity in the treatment of these infections.

MRSA bacteremia occurs more frequently in patients requiring intensive care and central line placement and is associated with mortality rates of 15-60% [1]. In a recent systematic review, MRSA bacteremia continues to have a significantly higher mortality rate than methicillin-susceptible *S. aureus* (MSSA) bacteremia [3]. Interestingly, though, in very low birth weight infants, MRSA and MSSA bacteremia have shown to have equivalent mortality [4]. Although MRSA bacteremia is associated with high treatment failure rates in adults [5,6], the epidemiology, clinical outcomes, and risk factors for treatment failure in children are limited [6].

To build on this topic within the field of paediatrics, we present a case of an infant with an unusual presentation of MRSA bacteremia. We also examine the clinical management of MRSA bacteremia in this patient, discuss the utility of ultrasound in reaching a diagnosis, and highlight risk factors for considering this diagnosis in this unique population.

## Case presentation

**Figure 1:**
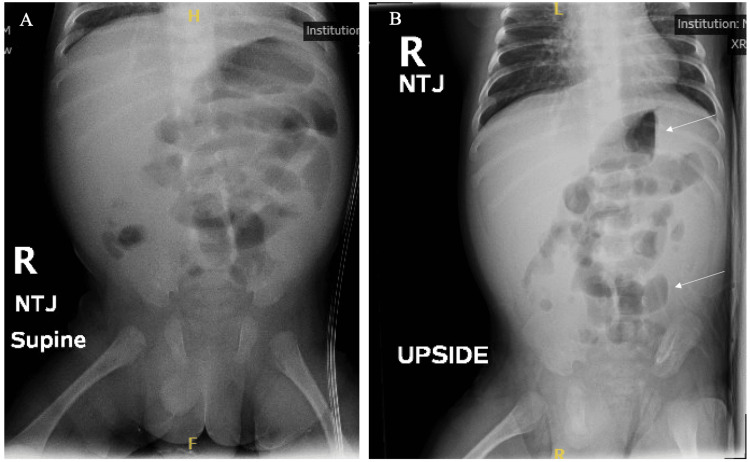
Abdominal X-rays. Initial supine (A) and lateral decubitus (B) abdominal x-rays demonstrated mildly distended proximal bowel and air fluid levels in a pattern suggesting ileus, enteritis, or possible developing obstruction.

A six-week-old twin male infant presented to the emergency department (ED) with a one-day history of bilious vomiting, decreased oral intake, and a firm abdomen. His birth history was significant for cesarean section at 33 weeks’ gestation due to placental abruption, and although he required the neonatal intensive care unit (NICU), his three-week stay was uneventful without the need for umbilical catheterization. On physical examination, he was febrile, tachycardic, and sleepy but appropriately responsive to exam. His abdominal exam showed a soft, mildly distended abdomen with normal bowel sounds. He grimaced and guarded with palpation. No hepatosplenomegaly was noted. Initial supine and decubitus abdominal X-ray demonstrated mildly distended proximal bowel in a pattern suggesting ileus, enteritis, or possibly a developing obstruction (Figure 1).

**Figure 2:**
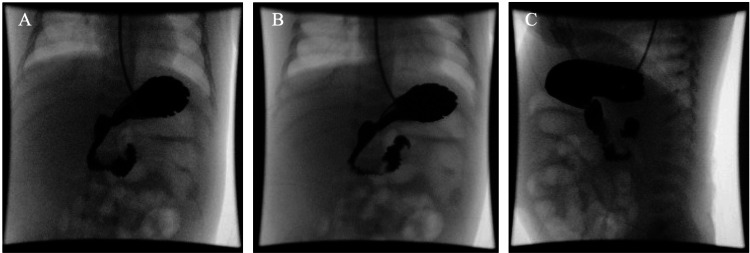
Fluoroscopic upper gastrointestinal series revealing no abnormalities. (A) In the frontal view, the duodenojejunal (DJ) junction crosses midline to the left of the left-side vertebral body pedicle. (B) In the frontal view, the DJ junction lies in line with the duodenal bulb. (C) In the lateral view, the second and third segments of the duodenum are located posteriorly in a retroperitoneal position. Images compared with normal findings reported by Walizai et al [7].

Due to the concern for malrotation and obstruction, a fluoroscopic upper gastrointestinal (GI) series was done; however, it was normal (Figure 2).

On presentation, laboratory investigation was significant for high-normal white blood cell count, elevated platelet count, elevated C-reactive protein (CRP), elevated procalcitonin, and elevated lactate (Table 1). The infant had a normal urinalysis and sterile urine culture. Additionally, the infant was evaluated for sepsis, in which two blood cultures yielded methicillin-resistant *Staphylococcus aureus *(MRSA). Cerebrospinal fluid studies were unremarkable, including a sterile CSF bacterial culture. Laboratory values downtrended with the initiation of antibiotics (Table 1).

**Table 1:** Laboratory Investigation.

Laboratory investigation	On presentation	2 days post-presentation	19 days post-presentation	Reference range
White blood cell (WBC) (/µL)	19,200	13,500	-	5.0-19.5x10^3^
Platelet count (/µL)	521,000	484,000	-	142-508x10^3^
C-reactive protein (CRP) (mg/dL)	16.3	13.7	0.7	<1.0
Procalcitonin (ng/mL)	45.8	8.3	-	<0.5
Lactate (mmol/L)	3.3	-	-	0.5-2.2

**Figure 3:**
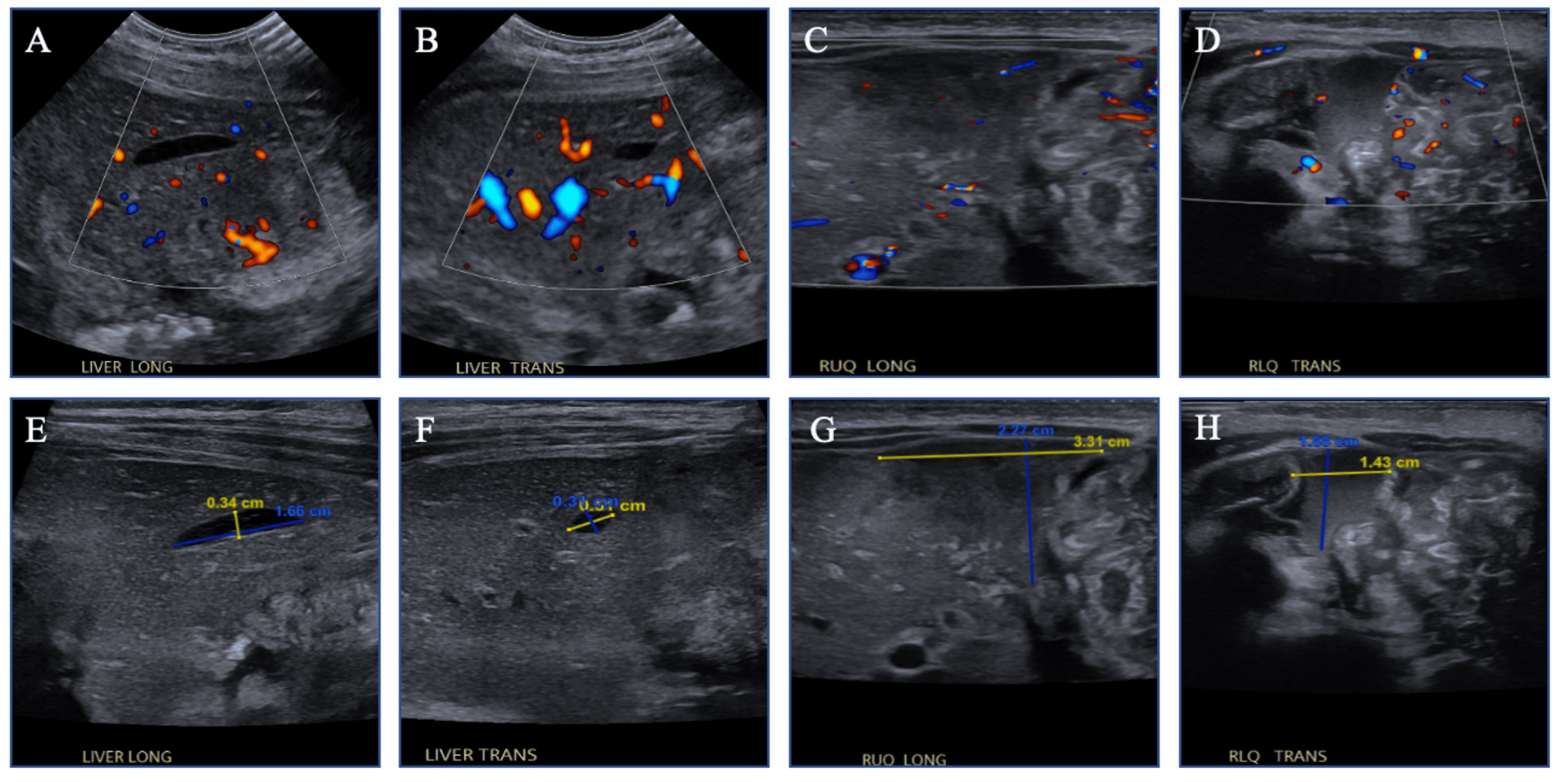
Abdominal ultrasound demonstrated subcapsular and intrahepatic abscesses with mass effect on the left lobe of the liver as well as on adjacent bowel loops and diffuse bowel wall thickening. (A) Liver long axis; (B) liver transverse axis; (C) right upper quadrant long axis; (D) right lower quadrant transverse axis views with Doppler showing vasculature; (E) liver long axis; (F) liver transverse axis view showing intrahepatic abscesses; (G) right upper quadrant long axis view demonstrating mass effect on left lobe of liver and adjacent bowel loops and diffuse bowel wall thickening; (H) right lower quadrant transverse axis view showing diffuse bowel wall thickening and complex free fluid in the peritoneal cavity.

With positive blood MRSA cultures, an abdominal ultrasound was performed to investigate a source of infection. It showed subcapsular and intrahepatic abscesses with mass effect on the left lobe of the liver and adjacent bowel loops, with diffuse bowel wall thickening and complex free fluid in the peritoneal cavity (Figure 3).

**Figure 4:**
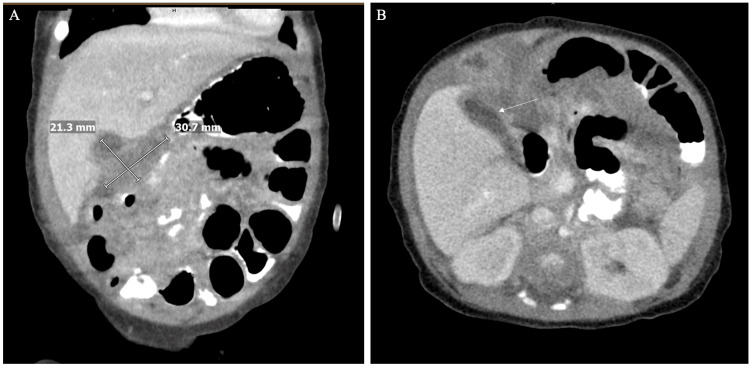
Computed tomography of the abdomen and pelvis with IV and oral contrast. (A) Coronal view and (B) axial view confirming liver abscesses with surrounding fluid collections and inflammatory changes.

Further characterization with computed tomography of the abdomen and pelvis with intravenous and oral contrast confirmed the abscesses with surrounding fluid collections and inflammatory changes (Figure 4).

The patient initially received intravenous ampicillin and ceftriaxone per guidelines at our institution for a febrile infant presenting at six weeks of life, pending sepsis and cerebrospinal fluid evaluation. Vancomycin was added when blood cultures yielded MRSA. Because of concern for peritonitis, ampicillin-sulbactam was provided but discontinued as abdominal examination and oral feedings improved. Given the patient’s clinical stability and clinical improvement on antibiotic therapy, invasive surgical intervention was not recommended. In addition to vancomycin, the infant also received 3 days of dual MRSA coverage therapy with daptomycin while awaiting blood sterilization. Once blood sterilization was achieved, he was transitioned to IV clindamycin monotherapy. The mean inhibitory concentration for clindamycin was 0.25 µg/ml, and since the erythromycin MIC was ≥8 µg/ml, a D-test was performed, which did not detect inducible clindamycin resistance. 

**Figure 5:**
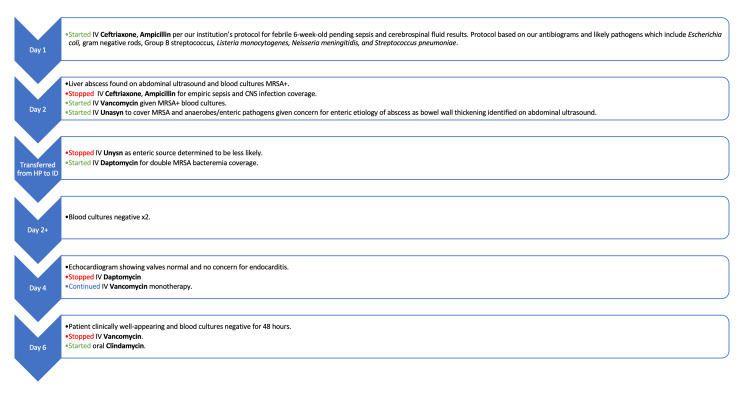
Flow chart demonstrating initiation and discontinuation of antibiotics in relation to infant's hospital course. *HP: hospital pediatrics service;  *ID: infectious disease service.

**Figure 6:**
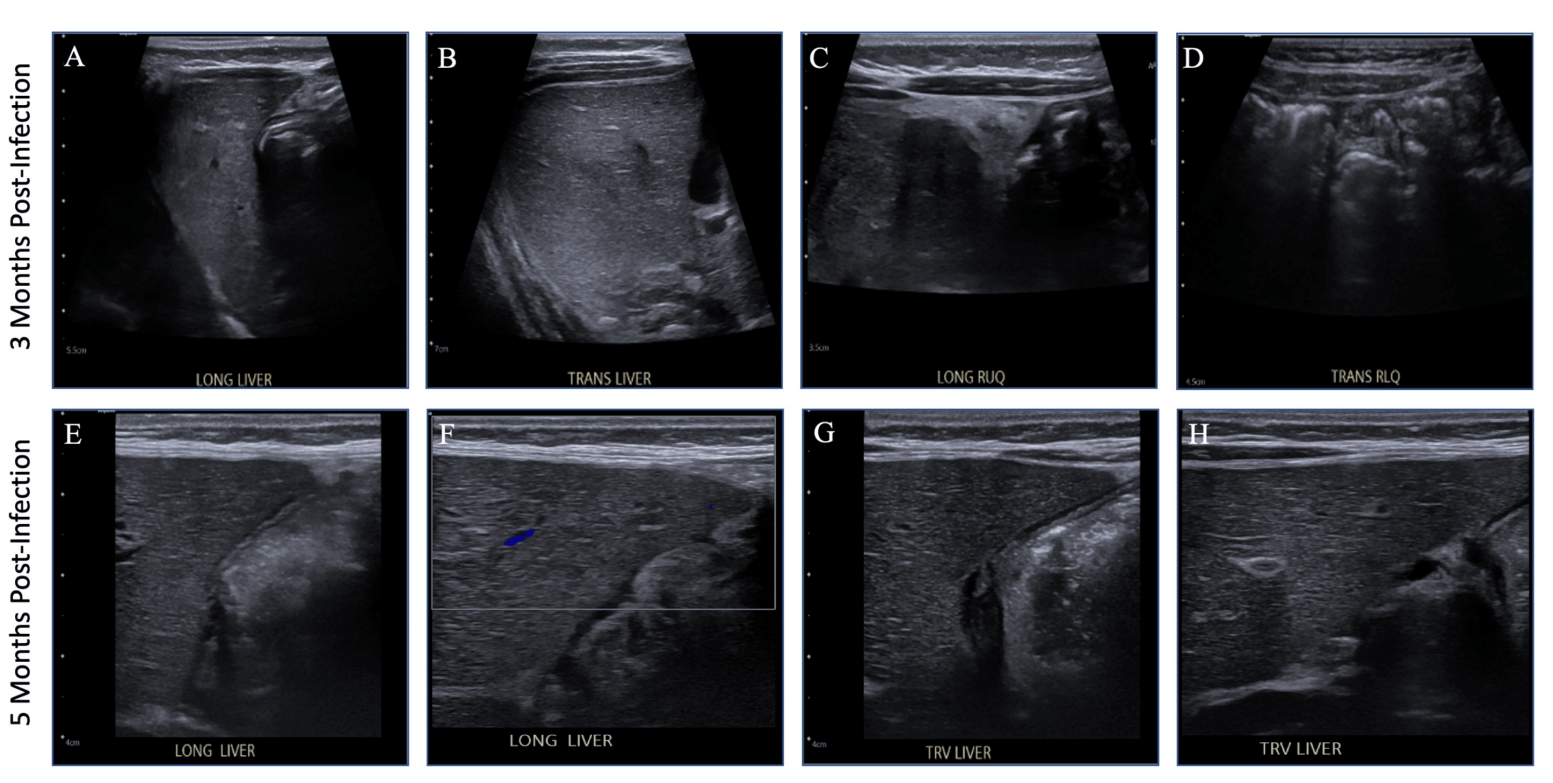
Repeat ultrasound three and five months post-infection. At three months post-infection, (A) liver long axis;  (B) liver transverse axis; (C) right upper quadrant long axis; (D) right lower quadrant transverse axis, views demonstrating near complete resolution of hepatic abscesses. At five months post-infection (E, F) liver long axis; (G, H) and liver transverse axis views demonstrating complete resolution of hepatic abscesses.

Repeat ultrasound showed decreased size of the abscess and resolution of inflammatory bowel changes. Immunodeficiency evaluation was also performed, and was notable for appropriate immunoglobulin concentrations and oxidative burst. On the ninth hospital day, the infant was discharged on oral clindamycin, which was then discontinued after six weeks of antibiotic therapy when the CRP normalized and abdominal ultrasound showed resolution of the abscess (Figures 5, 6, Table 1).

## Discussion

*S. aureus* is an infrequent cause of bacterial sepsis in young infants [8]. Currently, data are limited on optimal management [9]. Since there are no randomized controlled trials to inform treatment duration or the value of antibiotic combination therapy for *S. aureus* bacteremia in children, clinical management is based on expert opinion, guidelines, and historical practice [9]. With a positive *S. aureus* blood culture, initial treatment with an intravenous beta-lactamase-resistant beta-lactam agent (e.g., nafcillin) and vancomycin is recommended while awaiting antibiotic susceptibilities [9]. In the setting of MRSA-positive blood cultures, treatment with intravenous vancomycin or daptomycin is recommended [9]. However, there is expert debate that combination therapy with vancomycin and daptomycin may be beneficial in reducing toxin production [9]. In our case, the infant was treated with ampicillin and ceftriaxone for the usual community-acquired pathogens, prior to knowledge of the bacterial culture result. Once blood cultures yielded MRSA, vancomycin was started, with daptomycin added due to persistently positive blood culture results.

Central to MRSA management, a vancomycin trough level between 10-20 micrograms/millilitre is recommended for patients with serious infection. A vancomycin trough of 13.7 was achieved in our patient prior to discontinuing. Antibiotic therapy was changed to intravenous clindamycin once susceptibility results became known, with subsequent transition to oral clindamycin once the blood culture was sterilised. Given that source control was not achieved by surgical drainage of the abscesses, he was treated for a total antibiotic duration of 6 weeks when there was ultrasonographic resolution of the liver abscess.

Another important aspect of this case is the use of ultrasound in finding the source of infection and as a measure of adequate treatment. Ultrasound does not produce ionizing radiation, making it an ideal imaging modality in the pediatric population [10]. Furthermore, the ability to capture real-time images, its non-invasive nature, relatively low cost, and no requirement of sedation also offers favorable benefits compared to other imaging modalities [10]. However, it is important to recognize that ultrasound, as an imaging modality, has inherent limitations, particularly its reliance on adequately trained and experienced operators and interpreters, as well as its variable availability across institutions. On presentation, the infant had bilious vomiting and abdominal guarding, raising the possibility of malrotation and obstruction; however, the fluoroscopic upper GI series study was normal. Despite this, the presence of MRSA bacteremia with bilious emesis raised concern for intra-abdominal infection, and the use of abdominal ultrasound in this case was a convenient, safe, and effective modality in localizing the source of infection. Furthermore, serial abdominal ultrasounds were used to monitor the efficacy of treatment both in the hospital and in the outpatient setting. Although uncommon, this case demonstrates the utility of ultrasound in the diagnosis and clinical management of the hepatic abscess [11].

Across NICUs worldwide, *S. aureus* continues to be problematic [12]. Lower birth weight, younger gestational age, invasive procedures, and indwelling vascular catheters make critically ill neonates in the NICU particularly vulnerable [12,13]. Furthermore, these risk factors have been associated with MRSA colonization and disease [12,13]. Infants in the NICU may be colonized with MRSA from colonized parents, healthcare workers, and contaminated hospital surfaces [12,13]. To combat this risk, some NICUs employ screening and isolation protocols to minimize transmission [13]. While our patient spent 3 weeks in the NICU after birth, he was never screened for MRSA, and its origin remains unknown. As a result, it is important to consider MRSA infection on the differential especially among patients with a significant birth history and NICU stay.

## Conclusions

Our case demonstrates a rare manifestation of MRSA infection, namely a hepatic abscess, in which the initial presentation suggested an intestinal obstruction. The intestinal compression from a hepatic abscess and septic ileus masqueraded as a surgical abdomen. Invasive infection with MRSA is significant for its high morbidity and mortality. While high MRSA treatment failure rates are common in adult patients, outcomes are not well understood in children. As a result, we highlight this case to demonstrate successful medical management of MRSA bacteremia with hepatic abscess achieved by optimal MRSA antibiotic coverage using initial combination therapy and then monotherapy. Additionally, we establish the value of abdominal ultrasound in both the diagnosis and management of this infant.

With the high prevalence of multi-resistant staphylococcal invasive infections, the possibility of MRSA infection should be considered in patients with associated risk factors such as NICU hospitalization. While this patient’s NICU stay was overall uneventful and he did not require umbilical catheterization, MRSA colonization is possible or even likely to have occurred. We propose that screening for MRSA may be useful in risk-stratifying these patients both in the NICU and after discharge to home. Further data are needed to understand the number needed to treat and cost effectiveness of MRSA screening in the NICU on a population level, but in this case, it may have helped explain this presentation. Alternatively, identification of risk factors for invasive staphylococcal infection could further aid in early recognition. Interestingly, the patient’s twin remained healthy, and it is unknown if he is colonized with MRSA. Overall, this case emphasizes the importance of a broad workup in an ill infant with vomiting as his initial presentation deviated from common illness scripts.
